# Cytokine-Rich Adipose Tissue Extract Production from Water-Assisted Lipoaspirate: Methodology for Clinical Use

**DOI:** 10.1089/biores.2016.0030

**Published:** 2016-09-01

**Authors:** Jenny Lopez, Outi Huttala, Jertta-Riina Sarkanen, Ilkka Kaartinen, Hannu Kuokkanen, Timo Ylikomi

**Affiliations:** ^1^ Department of Cell Biology, School of Medicine, University of Tampere, Tampere, Finland.; ^2^ Department of Plastic Surgery, Unit of Musculoskeletal Diseases, Tampere University Hospital, Pirkanmaa Hospital District, Tampere, Finland.; ^3^ FICAM, Finnish Centre for Alternative Methods, School of Medicine, University of Tampere, Tampere, Finland.; ^4^ Science Center, Pirkanmaa Hospital District, Finland.

**Keywords:** acellular biological matrices, adipose, angiogenesis and vasculogenesis, biomedical engineering, growth factors

## Abstract

Proper functioning wound healing strategies are sparse. Adequate vascular formation to the injured area, as well as replacement of the volume loss, is fundamental in soft tissue repair. Tissue engineering strategies have been proposed for the treatment of these injury sites. Novel cell-free substance, human adipose tissue extract (ATE), has been previously shown to induce *in vitro* angiogenesis and adipogenesis and *in vivo* soft tissue formation. This study reports the translation of ATE preparation from laboratory to the operating room (OR). ATE samples for this study were derived from adipose tissue obtained with the water-jet assisted liposuction technique from 27 healthy patients. The variables studied included incubation time (15, 30, and 45 min), temperature (room temperature vs. 37°C), and filter type to determine the optimal method yielding the most consistent total protein content, as well as consistent and high expression of adipose-derived growth factors and cytokines, including: vascular endothelial growth factor, basic fibroblast growth factor, interleukin-6, adiponectin, leptin, and insulin-like growth factor. Following the optimization, samples were produced in the OR and tested for their sterility. No significant differences were observed when comparing extract incubation time points or incubation temperature. Nonetheless, when studying the different filter types used, a syringe filter with PES membrane with larger filter area showed significantly higher protein concentration (*p* ≤ 0.018). When studying the different growth factor concentrations, ELISA results showed less variation in cytokine concentrations in the OR samples with the optimized protocol. All of the OR samples were tested sterile. The devised protocol is an easy and reproducible OR-ready method for ATE generation. As an attractive source of growth factors, ATE is a promising alternative in the vast field of tissue engineering. Its clinical applications include volume replacement as a complement to fillers and improvement of the permanence of fat grafts and wound healing, among other bioactive functions.

## Introduction

Currently, the major challenge in tissue engineering lies not only in the high costs of material production and safety of the biomaterial used but also most importantly in the lack of efficacy in promoting vascular formation and soft-tissue replacement.^1–4^ In particular, neovascularization induction is a major obstacle for developing tissue engineering strategies.^5–9^

Mature human adipose tissue, considered an endocrine entity on its own, is a known source of growth and differentiation promoting factors.^10–16^ These growth factors have the ability to induce proliferation, migration, and differentiation of various cell lines.^13–17^ Extensive functions and clinical applications of growth factors are well reported in the literature.^18–24^ Adipose tissue is an excellent source of adipose stem cells in addition to the adipose-derived growth factors,^11,25–30^ and human adipose tissue is easily available through liposuction.

We have previously developed an acellular bioactive extract from mature adipose tissue, proven to contain important adipose tissue cytokines, including vascular endothelial growth factor (VEGF), basic fibroblast growth factor beta (FGFβ), interleukin 6 (IL-6), insulin-like growth factor 1 (IGF-1), adiponectin, and angiogenin, among others.^31,32^ This extract demonstrates a unique capacity of inducing angiogenesis and adipogenesis.^31^

Animal experimental models indicated that, in combination with hydrogel, this adipose tissue extract (ATE) promoted neovascularization and soft tissue expansion. In addition, it was shown that the permanence of the effect of the extract remained for 9 months. Furthermore, during short- and long-term follow-up, no hypersensitivity or foreign body reactions were reported with human extract in rat experimental models.^32^

When aiming at using this autologous extract in clinical studies, the development of a straightforward and reliable method for operation room preparation is essential. ATE was previously produced from solid mature adipose tissue and shown to be bioactive *in vitro* and *in vivo*.^31,32^ In the current study, ATE was produced from lipoaspirate material. This simple surgical procedure and operating room (OR) preparation would make ATE an attractive source of multiple growth factors and cytokines with a plethora of future applications.^32^

*In vitro*, these growth factors have demonstrated the induction and differentiation of adipocytes, endothelial cells, keratinocytes, chondrocytes, and osteoblasts, among others involved in tissue regeneration.^31,33,34^ The potential of ATE for different applications holds value in areas where soft tissue formation and volume are required, especially in volume loss and potentially for optimizing fat grafting permanence.^32^

In this study, the optimal method of human fat extract preparation was investigated with different variables, such as incubation time, temperature, and filter type. These characteristics were considered key when transferring the methodology from the laboratory to the OR while meeting the clinical and surgical standards. The goal was to develop a protocol that meets clinical standards that allow the surgeon to prepare the ATE in the OR for immediate application and translate the ATE laboratory protocol for clinical therapeutic use.

## Materials and Methods

### Ethical aspects

The study was conducted according to the Declaration of Helsinki, the European Guidelines on Good Clinical Practice, and was approved by the Ethics Committee of the Pirkanmaa Hospital District, Tampere, Finland (R03058). The human adipose tissue samples were obtained from surgical operations with informed consents at the Tampere University Hospital, Tampere, Finland.

### Samples

Human adipose tissue samples were obtained through the water-assisted liposuction technique (body-jet; Human Med AG, Germany) and processed under sterile conditions. A total of 27 patients were included in the study. The main indications for liposuction in these patients were fat grafting and body contouring. Exclusion criteria for the donors were patients receiving hormonal therapy, after cancer ablative surgery, active cancer, recent chemotherapy treatment, and comorbidities that contraindicated the surgery. All surgeries were uneventful, and no complications were reported. All tested samples were from female patients, and their mean age, weight, and body mass index are listed in Table 1. Lipoaspiration was performed in the abdominal subcutaneous tissue in 76.2% of the patients, from the flank region in 19% and from the thigh in 4.7%.

**Table 1.** **Patient Demographics**

	Mean value	Range
Age	51.95	33–68
Weight	77.1	57–94
Body mass index	28.1	21–38

### Lipoaspiration procedure

Liposuctions were performed under general or spinal anesthesia using the water-assisted technique (body-jet, Human Med AG, Germany). Tumescent solution containing 1 mg of adrenaline and 250 mg of lidocaine per 1000 mL saline was infiltrated. The pressure of infiltration and suction was done at a range of 2 or 3 (50 or 70 bar). Lipoaspiration was performed under −400 mbar pressure. As a guide, range 2 is equivalent to 110 mL/min and a range 3 to 130 mL/min of tumescent jet emission. Adipose tissue collection was done under a sterile environment into a canister (LipoCollector, Human Med, Germany) and transferred to 50 mL syringes. The amount of fat aspirated for sampling varied from 40 to 100 mL per patient.

### Production of ATE in the laboratory

In the laboratory, ATE was produced by adding Ringer lactate (Baxter Healthcare Corporation, Helsinki, Finland) to the adipose tissue sample at an approximate ratio of 1:1 and then processed according to the different study variables. Incubation was performed at 37°C water bath or at room temperature (RT). The incubation times studied were 15, 30, or 45 min. After incubation, the samples were sterile filtered with different 0.2 μm pore size syringe filters (Acrodisc^®^ filter, polyethersulfone PES membrane [PALL Life Sciences, New York]; Minisart NML filter, cellulose acetate membrane [Sartorius AG, Germany]; Filtropur S Plus filter, cellulose acetate membrane [Sarstedt & Co, Germany]; and Millex GP filter, PES membrane [Merck, Millipore, Germany]). Once filtered, the ATE was stored at −20°C until sample analysis.

### Production of ATE in the OR

**FIG. 1.**
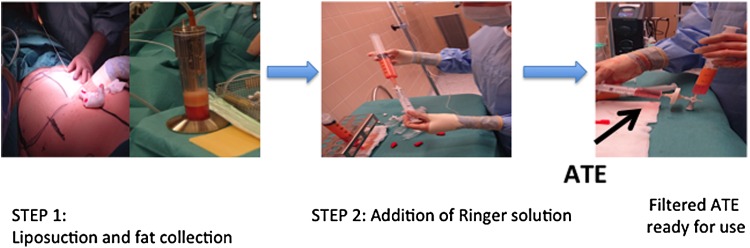
Steps for ATE preparation. Step 1, Liposuction and collection of adipose tissue with water-assisted liposuction. Step 2, Transfer of fat into syringes and addition of Ringer solution. Step 3, 30 min incubation and filtration to produce sterile ATE, which is ready for clinical use. ATE, adipose tissue extract.

ATE production was performed on a separate sterile bench in the OR. After performing the lipoaspiration, the adipose tissue was gently mixed with prewarmed (37°C) Ringer lactate solution at an approximate ratio of 1:1. The mixture was incubated for 30 min at RT. The lower layer containing the Ringer lactate solution was passed through a sterile filter and frozen at −20°C until further use. The preparation method of ATE is summarized in Figure 1. ATE samples have previously been shown to induce adipogenesis from 200 μg/mL upwards in cell culture.^31,32^ Thus, total protein concentration of 200 μg/mL was selected as the lowest acceptance limit of ATE samples. ATE samples prepared in the laboratory and OR originated from different donors.

### Sterility test of OR samples

Sterility test was performed from six ATE samples prepared in the OR. Approximately, 2 mL of ATE was added per BacT/ALERT system (bioMérieux SA, France) bottle. Aerobic BacT/ALERT SA and anaerobic bacteria testing BacT/ALERT SN (i.e., gram-positive and gram-negative bacteria and yeast) were performed for each ATE sample. Samples were cultured in BacT/ALERT 3D (bioMérieux SA) for 10 days before analyzing bacterial growth. “Negative” results in the system indicated that there was no bacterial or yeast growth.

### Measurement of protein concentration

Total protein content of the samples was measured using Pierce™ BCA Protein Assay Kit (Thermo Scientific, Waltham, MA) according to manufacturer's instructions using bovine serum albumin (BSA) as a standard. Results were measured after 30 min incubation at 37°C at 562 nm with Varioskan™ Flash Multimode Reader (Thermo Scientific).

### Measurement of growth factor concentration

Extract samples were tested with colorimetric sandwich ELISA, Custom made ELISA strips (Signosis^®^, Santa Clara, CA). The following cytokines were evaluated: VEGF, tumor necrosis factor alpha (TNFα), interferon gamma (IFNγ), granulocyte colony stimulating factor (G-CSF), granulocyte macrophage colony stimulating factor (GM-CSF), IL-6, IL-8, IGF-1, IL-1α, FGFβ, resistin, macrophage inflammatory protein (MIP-1), adiponectin, leptin, and rantes. Protein standards for custom human cytokine ELISA Strip (Signosis) were used with concentrations of 2 and 1 ng/mL. The ELISA strips were used according to manufacturer's instructions as follows. To each well, 100 μL of the studied ATE batch was added and incubated for 1 h with gentle shaking in RT. The liquid was then aspirated from each well and the wells were washed thrice with 200 μL of assay wash buffer per well. Subsequently, 100 μL of Streptavidin-HRP conjugate diluted 1:200 in diluent buffer was added to each well and incubated for 45 min at RT under gentle shaking. After incubation, the wells were washed thrice with 200 μL of washing buffer. One hundred microliters of substrate was added in each well and then incubated between 5 and 30 min per cytokine. The reaction was ended with the addition of 50 μL of stop solution to each well row simultaneously and detecting a visible color change of the standard. The optical density was determined at 450 nm with Varioskan Flash multimode reader (Thermo Scientific).

### Statistical analyses

Statistical analyses were performed and graphs processed with GraphPad Prism 5.0 (GraphPad Software, Inc., San Diego, CA). The results were reported as mean ± SD, and differences were considered significant when *p* < 0.05*, *p* < 0.01**, and *p* < 0.001***. Results of total protein concentration in incubation temperature comparison and laboratory versus OR production of ATE were analyzed with student's *t*-test and two-tailed posttest. The analyses of incubation time and filter type used were performed using One-way ANOVA with Tukey's posttest. The relationship of growth factor concentrations in OR samples to laboratory samples was calculated with Pearson's correlation and results depicted as *r* values.

## Results

### The effect of incubation temperature

**FIG. 2.**
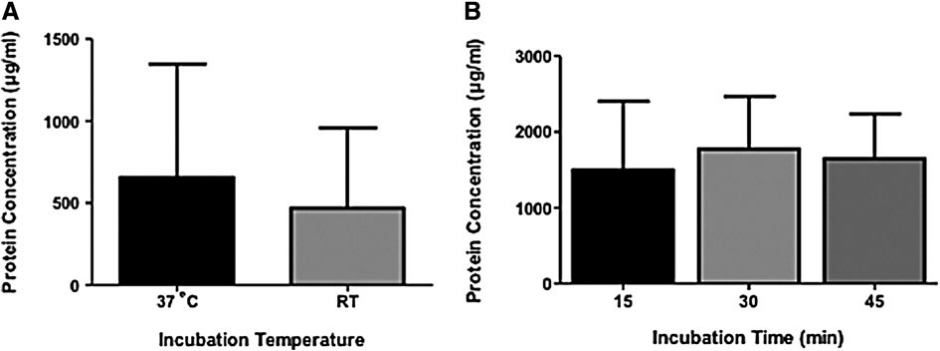
**(A)** Protein concentration obtained in 37°C and RT incubation. Comparison of total protein content of the ATE samples in different incubation temperatures. ATE was incubated for 30 min in 37°C or RT and filtered with cellulose acetate Filter 3. No significant difference was observed between incubation temperatures when statistically evaluated with student's *t*-test with two-tailed posttest (*p* 0.7207) and *n* = 3. **(B)** Protein concentrations obtained with 15, 30, and 45 min incubation. Comparison of total protein content of the ATE samples with incubation times of 15, 30, and 45 min. ATE was produced in equal conditions except for incubation time, that is, incubation was performed in RT and filtered with Filter 3. No significant difference was observed between incubation times when statistically evaluated with one-way ANOVA with Tukey's posttest (*p* 0.9923) and *n* = 3. RT, room temperature.

The effect of incubation temperature on ATE protein concentration was studied with a cellulose acetate membrane filter (Sarstedt, Germany) used in our previous *in vitro* studies.^31^ No significant difference in total protein concentration was observed between RT incubation and 37°C water bath incubation (Fig. 2A). However, there was a slightly higher concentration of total protein in water bath incubated samples compared to RT incubated samples.

### The effect of incubation time

Incubation times of 15, 30, and 45 min were studied to determine the shortest time for OR ATE extraction (considering possible immediate clinical use) in reference to protein yield. Incubation was performed in RT and the extract passed through a cellulose acetate membrane filter (Sarstedt, Germany).^31^ The total protein concentration between the time points did not differ significantly (multiplicity adjusted *p* ≥ 0.992, Fig. 2B), but the protein yield showed less variation and slightly higher values in the 30 min time point.

### Filter testing

**FIG. 3.**
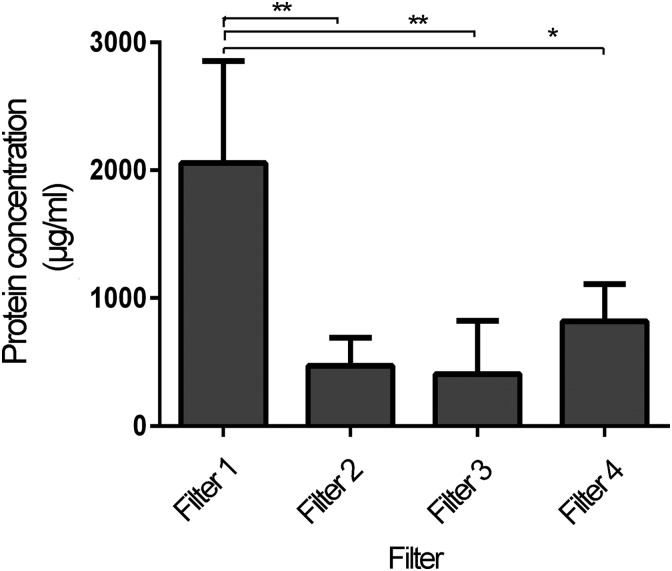
Protein concentrations obtained with four different filters. Comparison of total protein concentration when ATE was produced with 30 min incubation at RT using four distinct filters; PES Filter 1 (7.5 cm^2^), cellulose acetate Filter 2 (6.2 cm^2^), cellulose acetate Filter 3 (5.3 cm^2^), and PES Filter 4 (4.5 cm^2^). Statistical analysis was performed with One-way ANOVA with Tukey's posttest, *p* < 0.05* and *p* < 0.01** and *n* = 4.

To estimate whether the protein yield varied among different filter membranes, four different 0.2 μm filters were selected. ATE was produced at 30 min incubation at RT and subsequently strained through the filters. Close to 1000 μg/mL protein was obtained with all filters, but with polyethylene sulfone (PES) membrane and largest surface area, showed a significantly higher protein yield (multiplicity adjusted *p* ≤ 0.018) compared to the other filters (Fig. 3).

### Transfer of the extraction protocol from laboratory to the OR

**FIG. 4.**
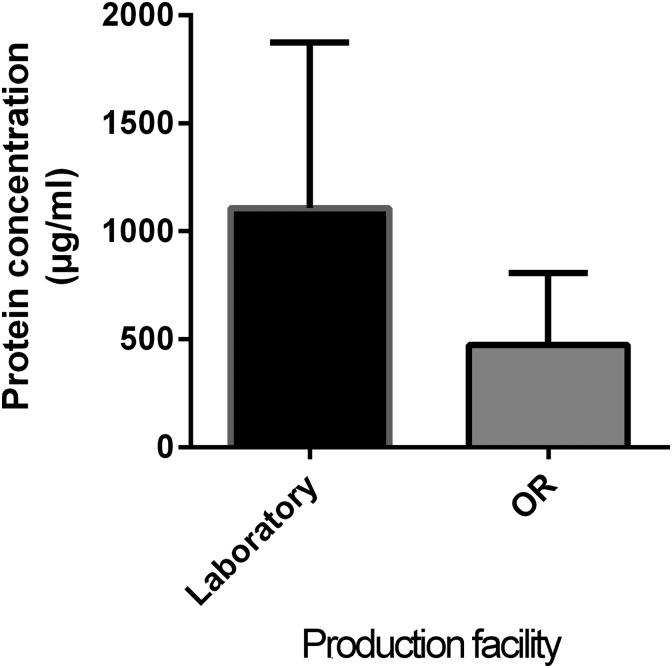
Protein concentrations obtained in OR versus laboratory. Comparison of total protein content between the ATE samples produced in laboratory and those produced in OR. ATE was incubated for 30 min in RT and filtered with PES Filter 1. No significant difference was observed between the total protein of the two production conditions as evaluated by student's *t*-test with two-tailed posttest (*p* 0.0922) and *n* ≥ 6. OR, operating room.

To transfer the laboratory methodology to the OR, ATE was produced at RT incubation for 30 min and subsequently filtered with PES membrane. The results in Figure 4 show that the samples produced in the laboratory had greater deviation yet higher protein concentrations. However, these were not significantly higher than the OR samples (*p* 0.0730, medians 1210 and 321.5, respectively).

### Growth factor content of the samples

**FIG. 5.**
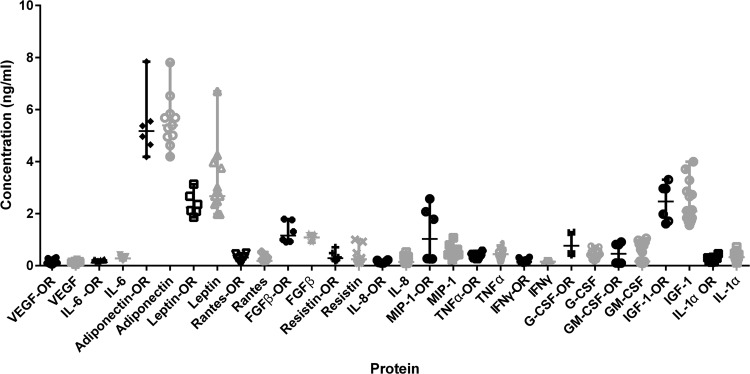
Specific protein concentrations in OR vs. laboratory. Analysis of 16 different adipokines in samples produced in laboratory in optimization phase versus samples produced in OR after optimization. Adipokines analyzed were VEGF, IL-6, adiponectin, leptin, rantes, FGFβ, resistin, IL-8, MIP-1, TNFα, IFNγ, G-CSF, GM-CSF, IGF-1 and IL-1α. Results depicted as mean ± SD, *n* ≥ 6.

To control the quality and assure the bioactivity of the samples, growth factor measurements of both laboratory and OR samples were studied. The results show that the growth factor yields were comparable between the samples produced in the laboratory and those from the OR (Fig. 5) although the correlations varied between the growth factors (Fig. 5). The samples had less variation when performed in the OR with the optimized protocol (Fig. 5). Most of the growth factors correlated well with laboratory samples and in addition, VEGF (also higher CV) and G-CSF produced higher mean growth factor concentrations in the OR with the optimized protocol.

### Sterility test

A sterility test was performed to ensure asepsis of the samples in the OR. All ATE samples were negative for bacterial growth in aerobic and anaerobic media.

## Discussion

One of the main challenges in tissue engineering is to discover a bioactive source of growth factors with regenerating properties while accelerating local action, enhancing volume replacement and stimulating the growth of multiple cell lines.^13,35–38^ The most important feature is to rapidly induce tissue neovascularization to avoid hypoxia and ischemia.^39–43^ Ideally, this bioactive material should also be cost beneficial, easy to prepare, and reproducible. In our previous studies, we have been able to isolate a novel cell-free bioactive substance, ATE, which not only induces angiogenesis and adipogenesis *in vitro* and in experimental models but also is rich in growth factors.^31,32^ The advantage of the current growth factor material is that as it does not contain cells, it should have less immune reactions and its allogeneic use could be possible. This would ease its clinical use. As ATE is a large mixture of growth factors, it has potential for better bioactivity. Clinicians are still struggling to improve adipose tissue transfer viability and working on ways to improve wound healing, scenarios that would benefit from growth factor use.

The main aim of the current study was to transfer the method of obtaining adipose tissue growth factors from the laboratory to the OR for later successful clinical use. ATE has previously shown to induce soft tissue formation when incorporated into hyaluronic acid and is therefore a very promising material for soft tissue replacement and soft tissue healing.^31,32^ The authors compared previously used parameters, protein yield, and growth factor content and focused on modifiable parameters when transferring the methodology from the laboratory to the clinic. The variables contemplated in this study were incubation time, incubation temperature, and filter type. During the translation process of ATE production from the laboratory to the OR, there are high clinical demands to be met not only from the clinical materials used but also to make this a simple method from surgical staff's point of view. Because in previous studies,^31,32^ whole solid fat was processed in the laboratory to obtain ATE, it was crucial to discover an easy method that would preserve an adequate protein yield.

This transfer method is not only important but also complicated and the impact of these variables had to be determined. Therefore, our aim was to maintain growth factor yields comparable to our previous studies, in a repeatable scenario. Moreover, our original studies were performed with solid adipose tissue that underwent processing, so we verified that the resulting ATE would, in fact, yield similar results when originating from lipoaspirates. As a lipoaspiration method, we used the water-assisted technique, which has been standardly used in our clinic due to its simplicity, and demonstrated adipose tissue viability preservation.^44^ Although the technique uses the power of water at certain pressures, it is gentle enough to preserve adipocytes with minimal tissue injury or blood loss. In addition, patient recovery is quicker and morbidity lower than traditional liposuction.^45,46^

In the current study, we optimized the preparation protocol of ATE to translate the production from the laboratory to the OR, studying three main variables. To begin with, incubation time is important considering the time the patient was in surgery under local, spinal, or general anesthesia. In ideal conditions, surgical time should be as short as possible to prevent patient perioperative complications and this is the reason why the time points of 15, 30, and 45 min were selected. Furthermore, because original ATE samples were incubated either in water bath or 37°C incubator, we found that comparing it to RT would offer the surgeon an easier method to produce ATE. In relation to the filter, during the translation process, we noticed that operating theaters use a variety of materials of high clinical standard demands. In our laboratory studies, we used filters that were not applicable to the OR environment; therefore, the challenge was to test those that did meet these standards while preserving the protein yield. The protocol was tested with four different filters with PES and cellulose acetate membranes. We noticed that although a larger filter area played a role, it seemed that the hydrophilic low-protein binding nature of the PES filter demonstrated advantages. Different to previous publications, we used lipoaspirate material that contains particles that easily clog filters, which also led us to choose the adequate PES filter type. However, we recognize that interpersonal variations play a great influence in the final protein yield. The optimization showed to reduce the variation between the samples and the specific growth factor concentrations were not lost during the protocol modifications. In addition, the samples produced in the OR passed the sterility tests performed.

By studying the effect of incubation temperature (RT vs. 37°C) and time (15, 30, and 45 min), we showed that the protocol is flexible enough to be performed in the busy OR setting without compromising the quality of the product.

When studying the protein yield, we observed that adequate amounts were obtained in short incubation times (15, 30, and 45 min); yet we settled for 30 min as slightly higher protein concentrations were achieved from this time point onward. Interestingly, temperature variations did not seem to affect total protein and cytokine yields. We noticed that although the filter characteristics may play a role, sample handling had an important effect on the protein yield. Samples that had to be neglected from the study (protein concentration <200 ng/mL, four samples) were performed in the laboratory, and thus, the time from the surgery to the preparation of ATE varied. This was based on previous studies where bioactivity was proven above this concentration of protein.^31,32^ Nonetheless, the filter material or surface area of the filter may also have an impact on the protein concentration and, therefore, on the feasibility of the ATE production procedure.

We observed lower variations in growth factor and cytokine measurements in the samples produced in OR compared with the laboratory samples. It is well known that interindividual differences in cytokine concentrations from freshly prepared primary cells are usually much higher than the variations seen with repeated preparations of the same donor.^47^ There is an inevitable patient-related variation due to the effects of body mass index, age, weight, comorbidity, and liposuction site that may affect protein yield, but the optimization of the protocol was successful in decreasing the sample-to-sample variation. The growth factor results are also in concordance with our previous studies at 1-h incubation.^32^

Since ATE is prepared from autologous adipose tissue, theoretically there are no risks for disease transmission, immunogenic reactions, or cancer. This solution is eluted from the filter, providing a cell-free enriched fraction of biologically active mediators (VEGF, IGF-1, etc.), most of which are key in wound healing. The most abundant adipokines released by adipocytes, leptin, and adiponectin,^48,49^ as well as IGF-I, were also predominant in this study. Inflammation-related factors observed by other investigators included leptin, resistin, adiponectin, TNFα, IL-6, and plasminogen activator inhibitor-1.^50^ In obese individuals, there is an increased expression of pro-inflammatory cytokines such as TNFα, TGFβ, and IL-6.^51^ In our previous studies, FGFβ, IGF-I, and IL-6 seemed to suggest the adipogenic and angiogenic potential of ATE.^31,32^ VEGF initiates angiogenesis,^52^ while IGF-1 and FGFβ promote wound healing.^53^ However, determining the optimal growth factor cocktail for adipogenesis and angiogenesis is challenging. Therefore, both the total protein concentration measurement and a set of growth factors measured should be kept as a quality assurance method also in further studies regarding ATE.

It is the authors' belief that the main point of this study was to conserve the growth factor yield when transferring from a laboratory methodology to an OR setting. Variations in growth factor concentrations are multifactorial, but we believe that interpatient variations play an important role. Therefore, we focused on maintaining protein yield compared to previous publications^31,32^ that demonstrated proven biological activity.

The protocol optimization showed that ATE is easily produced in the OR with a simplified method that preserves sterility and lays the foundation to implement ATE into the clinical setting. One of the major goals of this development process was to find acceptable range of variability for an optimal ATE preparation, without compromising the time pressure of a surgical setting. A distinct advantage of ATE is that it not only is prepared freshly from each patient for immediate use (e.g., treat wounds) but also that it can be frozen and used for sequential treatments. This eliminates the need for consecutive surgeries substantially reducing the surgeon's workload and also increasing patient comfort. ^47^ Material OR requirements for ATE preparation are minimal and the need for centrifugation or activation is bypassed. The fact that sufficient protein content can be obtained in an even shorter incubation time than studied earlier^32^ and under RT makes ATE a more attractive choice for clinicians compared to currently used products.

Orthobiologics is a relatively new science that involves application of naturally found materials from biological sources (e.g., cell-based therapies) and offers exciting new possibilities to promote and accelerate bone and soft tissue healing.^54,55^ The goal of this discipline is to enhance the body's innate ability to repair and regenerate.^55^ In fact, the inductive microenvironment has previously shown to enhance migration of adipose stromal cells in the surrounding tissue and stimulate the cells to differentiate into mature adipocytes.^56^ Growth factors are known to play essential roles in wound repair and may hold the key to successful healing. However, the use of single cytokines has generated disappointing results compared to a combination of these.^47,57^ Therefore, it is evident that wound healing is a very complex process that relies on the harmonious action of a myriad of growth factors. This is a fundamental principle in wound healing.^47^ The use of secretory factors from adipose tissue to influence the wound microenvironment may be a feasible approach to develop topical applications for quicker repair.^58^ We suggest the use of adipose tissue secretome to produce an extract rich in cytokines for topical application of wounds, rather than using the difficult process of enriching the patients' stem cells *in vitro*.^58^ Burdensome steps, including the use of collagenase for digestion of tissue to isolate stem cells, would be obviated when generating ATE, an important advantage in the clinical setting.^58^ To date, adipose tissue is already used as an active bio dressing to treat wounds with promising wound healing results.^58,59^ Similarly, adipose tissue and its secretome have positively influenced wound healing and tissue regeneration.^60,61^ In fact, the speed and quality of wound healing have been enhanced significantly in wounds treated with adipose tissue-derived factors.^58,60^ *In vitro*, the conditioned medium of adipose tissue composed of a multitude of adipokines and growth factors, proved to potently induce the proliferation of adipose stem cells and endothelial cells, comparable to the conditioned medium of stem cells.^59,62^

The potential clinical use of ATE is currently under study in different clinical applications where growth factor may be required to enhance tissue vascularization, healing, cell migration, growth, and proliferation. The use of ATE would grant the benefits of a cell-free approach and would, therefore, have a more extended scope, not just limited to autologous application.^58^ Furthermore, it would make the production of an off-the-shelf product according to good manufacturing practice much easier. Especially for the potential clinical applications of adipose tissue, the effect of its secretome needs to be investigated thoroughly.^58^ In addition, the proangiogenic effect reported in this study may pose as an attractive starting point for investigating the potential impact of adipose tissue secretory factors on neovascularization in several tissue regeneration applications.

## Conclusion

ATE is a very promising bioactive agent for a plethora of clinical uses. The relative ease of preparation, applicability in the clinical setting, favorable safety profile, and possible beneficial outcome make ATE a promising therapeutic approach for future regenerative treatments. It is easy to obtain, inexpensive, and by being autologous and cell-free, it is minimizing common problems of tissue engineering. Its proven adipogenic and angiogenic properties, along with being an abundant source of growth factors, make ATE an appealing microenvironment for cell proliferation, migration, and differentiation. Under these circumstances, any clinical scenario in need of tissue volume addition, wound healing, and adequate vascularization would benefit from ATE. The consistent protein and growth factor concentrations prove the repeatability of the method, and thus, the extract production method can be successfully produced in the OR environment and is now ready for clinical research.

## Abbreviations Used

FGFβ: fibroblast growth factor beta
G-CSF: granulocyte colony stimulating factor
ATE: human adipose tissue extract
IGF-1: insulin-like growth factor 1
IL-6: interleukin 6
OR: operating room
PES: polyethylene sulfone
TNFα: tumor necrosis factor alpha
VEGF: vascular endothelial growth factor
